# Peroxisomal ACBD4 interacts with VAPB and promotes ER-peroxisome associations

**DOI:** 10.1080/15384101.2017.1314422

**Published:** 2017-05-02

**Authors:** Joseph L. Costello, Inês G. Castro, Tina A. Schrader, Markus Islinger, Michael Schrader

**Affiliations:** a Department of Biosciences, University of Exeter, Exeter, UK; b Institute of Neuroanatomy, Center for Biomedicine & Medical Technology Mannheim, Medical Faculty Mannheim, University of Heidelberg, Mannheim, Germany

**Keywords:** ACBD4, ER, membrane contact sites, Peroxisomes, VAPB

## Abstract

Cooperation between cellular organelles such as mitochondria, peroxisomes and the ER is essential for a variety of important and diverse metabolic processes. Effective communication and metabolite exchange requires physical linkages between the organelles, predominantly in the form of organelle contact sites. At such contact sites organelle membranes are brought into close proximity by the action of molecular tethers, which often consist of specific protein pairs anchored in the membrane of the opposing organelles. Currently numerous tethering components have been identified which link the ER with multiple other organelles but knowledge of the factors linking the ER with peroxisomes is limited. Peroxisome-ER interplay is important because it is required for the biosynthesis of unsaturated fatty acids, ether-phospholipids and sterols with defects in these functions leading to severe diseases. Here, we characterize acyl-CoA binding domain protein 4 (ACBD4) as a tail-anchored peroxisomal membrane protein which interacts with the ER protein, vesicle-associated membrane protein-associated protein–B (VAPB) to promote peroxisome-ER associations.

## Introduction

The study of organelle interactions at membrane contact sites is an area of cell biology which has expanded rapidly over the last decade due to the understanding that interorganellar communication is vital for cellular function. A striking example of the importance of organelle interplay is found in the relationship between the endoplasmic reticulum (ER) and peroxisomes (reviewed in ref. 1). These 2 organelles have been known to be intimately associated since ultrastructural studies in the 1960s detected close apposition between ER tubules and peroxisomal membranes.^2,3^ A number of metabolic pathways require the combined action of both peroxisomal and ER-resident enzymes. Most notably in the production of ether-phospholipids such as plasmalogens which requires generation of a characteristic ether bond by peroxisomal enzymes before the remaining steps in biosynthesis can be completed in the ER.^4^^,^^5^ Failure to properly assemble peroxisomes (e.g. in Zellweger spectrum disorders),^6^ mutations in the genes which encode the peroxisomal enzymes or import factors which bring the enzymes into peroxisomes result in a deficiency in ether phospholipid production and lead to diseases such as rhizomelic chondrodysplasia punctata (RCDP).^7-9^ In mammals, as well as linking with peroxisomes for metabolic cooperation, the ER can also play a role, perhaps in collaboration with mitochondria, in the *de novo* generation of peroxisomes.^10-12^ The full extent the ER plays in peroxisome biogenesis is unclear but appears to at least involve the provision of membrane phospholipids (and potentially membrane proteins such as Pex16) for formation of the peroxisomal membrane.^13^^,^^14^

As well as interacting with peroxisomes, the ER forms contact sites with mitochondria, Golgi complex, plasma membrane, and endosomes^15-17^ (see ref. 18 for a comprehensive list). Two key players are vesicle-associated membrane protein-associated proteins – A and B (VAPA/B), which are present in several important contact sites involving the ER.^19^ VAPA/B are ER-resident membrane proteins containing a major sperm protein (MSP) domain that interacts with proteins containing a FFAT or FFAT-like motif.^20^ One such protein is PTPIP51, a mitochondrial membrane protein which interacts with VAPB to mediate mitochondria-ER associations, facilitating calcium exchange and regulating autophagy.^17^^,^^21^

Recently, we identified peroxisomal acyl-CoA binding domain protein 5 (ACBD5) and VAPB as interaction partners of a molecular tether which physically links peroxisomes to the ER in mammals.^22^ Both VAPB and ACBD5 are C-tail-anchored (TA) membrane proteins, defined as proteins which contain N-terminal functional domains followed by a single transmembrane domain (TMD) close to the C-terminus and a short C-terminal tail region. These characteristic properties dictate that TA proteins are post-translationally sorted to their target membrane with the N-terminus facing the cytosol.^23^ In another recent study we investigated the targeting properties of TA proteins, discovering the importance of interplay between TMD hydrophobicity and tail-charge, and developed a statistical model to predict cellular localization of TA proteins based on these physicochemical parameters.^24^ Using this bioinformatics prediction tool we identified an isoform of ACBD4 (isoform 2), a predicted TA protein of unknown function and localization, as a potential peroxisomal protein and confirmed this by expression of Myc-ACBD4iso2 in COS-7 cells.^24^ ACBD4, like ACBD5, is a member of the ACBD family which is characterized by the presence of an acyl-CoA binding domain. Seven different ACBDs have been identified in mammals but the acyl-CoA binding protein structural fold has been found in 48 different protein architectures across all species.^25^ Thus, although ACBD4 and ACBD5 share 58% sequence identity this is mainly isolated to similarities in the N-terminal acyl-CoA binding domain, with the rest of the proteins showing significant differences.

Here, we show that ACBD4 isoform2 is a tail-anchored peroxisomal protein which interacts with the ER-resident protein VAPB to facilitate interaction between the 2 organelles. These results suggest that ACBD4, like ACBD5, can act as a molecular tether, physically linking peroxisomes and the ER making this the second protein involved in peroxisome-ER contacts in mammals.

## Results

**Figure 1:**
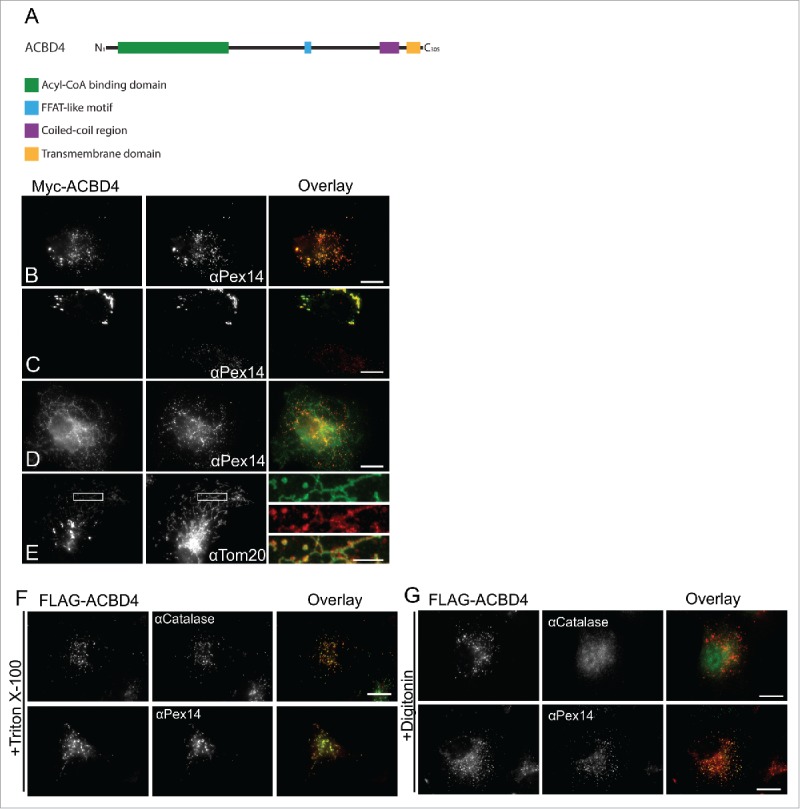
. ACBD4iso2 is a peroxisomal C-tail-anchored protein. (A) Schematic overview of ACBD4iso2 domain structure. ACBD = acyl-CoA binding domain, FFAT-like = 2 phenyalanines in an acidic tract, CC = coiled coil, TMD = transmembrane domain. (B-E) Subcellular localization patterns for ACBD4iso2. COS-7 cells transfected with Myc-ACBD4iso2 were immunolabelled using αPEX14 (peroxisomal marker), αTOM20 (mitochondrial marker) and αMyc antibodies. (E) Higher magnifications of boxed regions are shown (F-G) Differential permeabilisation. COS-7 cells expressing FLAG-ACBD4iso2 were fixed, permeabilised with either Triton X-100 (0.2% in PBS) (F) or digitonin (2.5µg/ml in PBS) (G), and stained with αCatalase (PO matrix), αPEX14 (PO membrane) or αFLAG antibodies. Bars, 10 µm (overlay), 2µm (magnified sections).

**ACBD4iso2 is a C-tail-anchored membrane protein which shows peroxisomal targeting when expressed in COS-7 cells.** ACBD4 has 3 major isoforms (as defined by UniProt identifier: Q8NC06) one of which, isoform 2 (UniProt identifier: Q8NC06–2), is predicted to contain a C-terminal TMD and tail. In addition to the characteristic N-terminal acyl-CoA binding domain, other predicted structural features in ACBD4iso2 include a potential coiled-coil domain and a predicted FFAT-like motif (Fig. 1A). Previously, we showed that Myc-ACBD4iso2 expressed in COS-7 cells localized to peroxisomes.^24^ Here, we further characterize ACBD4 localization showing that while we always observe Myc-ACBD4iso2 targeting to peroxisomes (Fig. 1B), when expression levels are high we observe changes in peroxisome morphology (Fig. 1C) and weak, non-peroxisomal signal (Fig. 1D) which co-localizes with a mitochondrial marker (Fig. 1E). This phenomenon has also been observed for other peroxisomal TA proteins such as Pex26^26^ which also shows mitochondrial localization when expression is high.

To confirm that ACBD4iso2 is a C-tail-anchored protein with the N-terminus exposed to the cytosol we performed differential permeabilisation experiments using either digitonin or Triton X-100. Triton X-100 permeabilises peroxisomal membranes whereas upon digitonin treatment peroxisome membranes remain intact.^27^^,^^28^ Accordingly, following digitonin treatment the peroxisomal matrix marker catalase was inaccessible to antibodies and was only detected after Triton X-100 treatment (Fig. 1F). After digitonin treatment the N-terminal FLAG-tag of FLAG-ACBD4iso2 was detectable using FLAG antibodies indicating that the N-terminus of ACBD4 is exposed to the cytosol (Fig. 1G) similar to what was found for ACBD5.^24^

**Figure 2:**
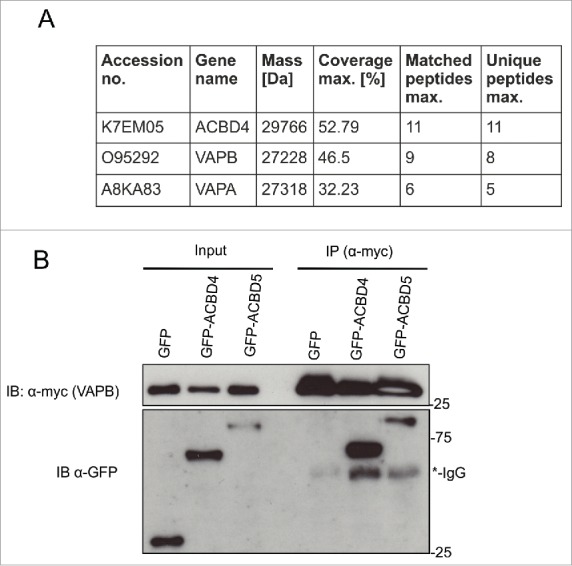
. ACBD4iso2 interacts with VAPB. (A) Identification of VAPB and VAPA by MS after co-immunoprecipitation (IP) with GFP-ACBD4iso2 from COS-7 cells (results from 3 experiments); GFP used as control. Only protein IDs which did not appear in any of the GFP only control experiments were considered. (B) Immunoprecipitation (IP) of GFP-ACBD4iso2 and Myc-VAPB after co-expression in COS-7 cells. GFP used as a negative control and GFP-ACBD5 as a positive control. Samples were immunoprecipitated (GFP-Trap) and immunoblotted (IB) using Myc/GFP antibodies.

**ACBD4iso2 interacts with VAPB.** As the function of ACBD4 is unknown we performed proteomics studies to identify potential binding partners. GFP-ACBD4iso2 and GFP alone were expressed in COS-7 cells and pull down studies and mass spectrometry (MS) analyses were performed in triplicate. Following filtering of the results (based on a previous study^29^ only protein IDs with >1 unique peptide hits, >20% peptide:protein coverage and overall MS scores >30, which did not appear in any of the GFP only control experiments, were considered) from MS experiments we identified the ER membrane proteins VAPA and VAPB as candidate binding partners (Fig. 2A). We next confirmed the ACBD4-VAPB interaction by immunoprecipitation (IP). GFP-ACBD4iso2 and Myc-VAPB were co-expressed in COS-7 cells and their interaction was assessed by IP using Myc-TRAP magnetic agarose beads (Fig. 2B). As a positive control we used GFP-ACBD5 which we had previously shown to interact with Myc-VAPB using the same assay.^22^ Using this assay we were able to confirm interaction between ACBD4iso2 and VAPB.

**Figure 3:**
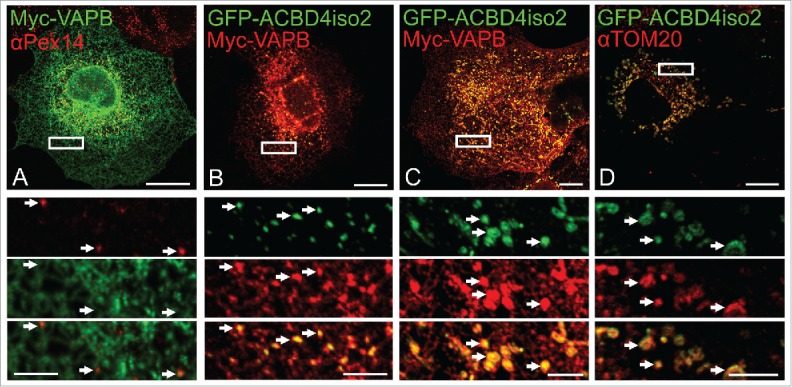
. ACBD4iso2/VAPB co-expression promotes PO-ER association. COS-7 cells were transfected with (A) Myc-VAPB alone (immunolabelled using αPEX14, a peroxisomal marker), (B) Myc-VAPB co-expressed with GFP-ACBD4iso2, (C) Myc-VAPB co-expressed with GFP-ACBD4iso2 showing mitochondrial mistargeting. (D) Co-localization of GFP-ACBD4iso2 with Tom20 (mitochondrial marker). Arrows highlight PO-ER association. Bars, 20 µm (overview), 5 µm (cut outs).

**Co-expression of GFP-ACBD4iso2/Myc-VAPB promotes ER-PO associations.** Having established that ACBD4 can interact with VAPB we wanted to test if ACBD4, like ACBD5, can play a role in mediating peroxisome-ER associations. To test this, we co-expressed Myc-VAPB and GFP-ACBD4iso2 in COS-7 cells and analyzed ER-PO localization using confocal microscopy (Fig. 3). In our previous study we observed that when both ACBD5 and VAPB were overexpressed we could observe increased ER-peroxisome associations which, strikingly, allowed vizualization of discrete peroxisomal structures when using VAPB as an ER marker.^22^ Here, this characteristic PO-ER association was also observed when ACBD4 and VAPB were co-expressed together but not individually (Fig. 3A, B). Furthermore, when we examined cells in which ACBD4 was found at mitochondria (see Fig. 1C) we detected increased association of VAPB-labeled ER with the mitochondrial marker, suggesting that in this case mis-targeted ACBD4 was mediating increased ER-mitochondria interactions (Fig. 3C). These findings support a role for ACBD4 and VAPB interaction in ER-peroxisome tethering.

### Discussion

**Figure 4:**
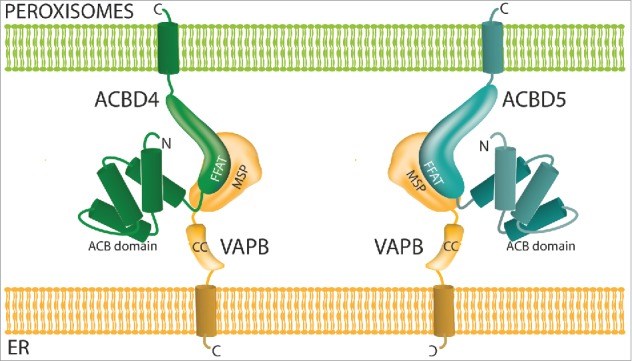
. Model of ACBD4/ACBD5-VAPB interaction. ACBD4 and ACBD5 are both C-tail anchored peroxisomal membrane proteins with functional domains in the cytoplasm which can interact with the MSP domain of ER-resident VAPB via a FFAT-like motif. ACB = acyl-CoA binding, FFAT = 2 phenyalanines in an acidic tract, MSP = major sperm protein binding domain.

The data presented here, namely that ACBD4 is localized to peroxisomes and interacts with the ER protein VAPB to promote ER-peroxisome associations support the assumption that ACBD4 is acting as a tether.^30^ In a recent publication we identified the first molecular mechanism which allowed peroxisome-ER interactions in mammalian cells via a tether consisting of peroxisomal ACBD5 and ER-resident VAPB^22^ (Fig. 4). We showed that in the absence of ACBD5/VAPB peroxisomal membrane expansion was reduced, suggesting that the lipid flow from the ER to peroxisomes required for peroxisomal membrane growth was disrupted. In addition the movement of peroxisomes was increased. Simultaneously the group of Peter Kim used a parallel approach to reach the same conclusions, additionally reporting that plasmalogen synthesis is impaired when the VAPB-ACBD5 tether is disrupted.^31^ Recent studies have now identified patients carrying pathogenic mutations which lead to the loss of ACBD5 protein.^32-34^ In these cases increased levels of very-long-chain fatty acids (VLCFAs) were detected in patient cells likely due to reduced import into peroxisomes. This suggested a role for ACBD5 in binding VLCFAs in the cytosol and facilitating their transport into peroxisomes which would then be mediated by the peroxisomal ABC transporters at the peroxisomal membrane.^35^^,^^36^ It is not clear if ACBD5 interacts with the ABC transporters, with a recent study failing to identify ACBD5 as an interacting partner of ABCD2,^37^ nor how the tethering function of ACBD5 is linked to its function in β-oxidation of VLCFAs. However, as ACBD4 also contains an acyl-CoA binding domain and a predicted FFAT-like motif it is tempting to speculate that ACBD4 may play a similar role to ACBD5 (Fig. 4). The differences between the 2 proteins may lie in substrate specificity, expression profile, regulation or type of tether. Yagita and colleagues^34^ reported that ACBD5 is able to preferentially bind VLCFAs *in vitro* but its optimal substrate was not identified and may differ from the optimal substrate for ACBD4. In our previous study^22^ knockdown of ACBD5 showed significant effects on the extent of peroxisome-ER interactions in HepG2 cells. As ACBD4 is reported to be expressed in these cells,^38^ it is unlikely that normal ACBD4 levels can fully complement the function of ACBD5. It is possible that ACBD5 is the major tether for peroxisome-ER contacts whereas ACBD4 may play a role in a more specialized ER-peroxisome association. The presence of more than one tether which can link peroxisomes and the ER is in line with the multiple different tether combinations used by other organelles to cater for specialized functions.^39^ Future studies will address these points and contribute to the understanding of the roles of ACBD4 and ACBD5 in peroxisome-ER interplay, lipid metabolism and how their dysfunction links to disease.

## Materials and Methods

### Plasmids and antibodies

Myc-VAPB plasmid was kindly provided by C. Miller (King's College London, UK). A human ACBD4iso2 cDNA clone (Cusabio Life Sciences, http://www.cusabio.com/Clone/ACBD4–158327.html) was used as a template to generate GFP-ACBD4iso2 and FLAG-ACBD4iso2 using eGFP-C1 and pCMV-2B vectors respectively. Primers: ACBD4_iso2_GFP_For = AAACTCGAGCTATGGGCACCGAG AAAGAAAGCCCAGAGCCCGAC, ACBD4_iso2_GFP_Rev = TTGGATCCTCACCTC TTTTGGGTCCGAAACATTCGGAAGAGCC (XhoI, BamHI digest into eGFP-C1).

ACBD4_myc_For = AAGGATCCATGGGCACCGAGAAAGAAAGCCCAGAGCCCGAC, ACBD4iso2_myc_Rev = CTCTCGAGTCACCTCTTTTGGGTCCGAAACATTCGGAAGA GCC (XhoI, BamHI digest into pCMV2B). Antibodies were as follows: polyclonal rabbit anti-PEX14 (kindly provided by D. Crane, Griffith University, Brisbane, Australia); anti-catalase (Abcam, http://www.abcam.com/catalase-antibody-ab88650.html); anti-GFP (Thermofisher, https://www.thermofisher.com/antibody/product/GFP-Tag-Antibody-Polyclonal/A-11122); anti-Myc (Abcam, http://www.abcam.com/myc-tag-antibody-ab9106.html); anti-FLAG (SIGMA, http://www.sigmaaldrich.com/catalog/product/sigma/f3165?lang = enandregion = GB).

### Cell culture and transfection

COS-7 cells (African green monkey kidney cells; ATCC, https://www.lgcstandards-atcc.org/products/All/CRL-1651) were cultured in DMEM, high glucose (4.5 g/L) supplemented with 10% FBS, 100 U/ml penicillin and 100 µg/ml streptomycin at 37°C with 5% CO_2_ and 95% humidity. COS-7 cells were transfected using diethylaminoethyl (DEAE)-dextran (Sigma-Aldrich, http://www.sigmaaldrich.com/catalog/product/sigma/d9885) as described.^40^

### Immunofluorescence and microscopy

Cells were processed for immunofluorescence 24 h after transfection as described previously.^41^ Cell imaging was performed using an Olympus IX81 microscope equipped with an UPlanSApo 100x/1.40 Oil objective (Olympus Optical, Hamburg, Germany), eGFP ET filter-set (470/40 ET Bandpass filter, Beamsplitter T495 LPXR and 525/50 ET Bandpass filter (Chroma Technology GmbH, Olching, Germany)), and TxRed HC Filter Set (562/40 BrightLine HC Beamsplitter HC BS 593, 624/40 BrightLine HC (Semrock, Rochester, USA)). Digital images were taken with a CoolSNAP HQ2 CCD camera and adjusted for contrast and brightness using MetaMorph 7 (Molecular Devices, https://www.moleculardevices.com/systems/metamorph-research-imaging/metamorph-microscopy-automation-and-image-analysis-software). Confocal images were obtained using a Leica SP8 equipped with: Argon laser (488), DPSS561 laser (561), HC PL APO 63x/1.3 Oil objective, HC PL APO 100x/1.44 Oil objective, Hybrid detectors (HyD).

### Immunoprecipitation

GFP-ACBD4iso2, or GFP only control, and Myc-VAPB were expressed in COS-7 cells. After 48 h cells were washed in PBS and lysed in ice-cold lysis buffer (25 mM Tris-HCl pH 7.5, 150 mM NaCl, 0.5 mM EDTA, 1% NP-40, 1 mM PMSF and mini protease inhibitor cocktail (Roche, http://www.sigmaaldrich.com/catalog/product/roche/11836170001)). Unsolubilised material was pelleted by centrifugation at 100,000 × g_av_. Clarified lysates were then mixed with Myc-TRAP magnetic agarose beads (ChromoTek, http://www.chromotek.com/products/nano-traps/myc-trapr/) and incubated for 2 h at 4°C. Beads were washed extensively with lysis buffer and bound proteins were either eluted with Laemmli buffer or further processed for mass spectrometry analysis. Immunoprecipitates and total lysates were analyzed by Western immunoblotting.

### Mass spectrometry (MS)

For MS analysis, immunoprecipitations (see above) from 3 independent experiments were analyzed for both GFP-ACBD4iso2 and a GFP only control. Sample preparation and protein identification were performed by the University of Bristol Proteomics Facility as described previously.^29^ Extracted MS/MS spectra were searched against the Uniprot Human database and were filtered at 5% FDR. Additional filtering parameters were based on a previous study.^29^ Only protein IDs with >1 unique peptide hits, >20% peptide:protein coverage and overall MS scores >30, which did not appear in any of the GFP only control experiments, were considered.

## Abbreviations

ACBD: acyl-CoA binding domain
IP: immunoprecipitation
PO: peroxisomes
VAPB: vesicle-associated membrane protein-associated protein–B
